# Efficient Treatment of Rheumatoid Arthritis by Degradable LPCE Nano-Conjugate-Delivered p65 siRNA

**DOI:** 10.3390/pharmaceutics14010162

**Published:** 2022-01-11

**Authors:** Xiaohua Chen, Bailing Zhou, Yan Gao, Kaiyu Wang, Jieping Wu, Ming Shuai, Ke Men, Xingmei Duan

**Affiliations:** 1 State Key Laboratory of Biotherapy and Cancer Center, West China Hospital of Sichuan University, Chengdu 610041, China; cxh_an@163.com (X.C.); edithgaoyan@outlook.com (Y.G.); wky17381492350@163.com (K.W.); wjp18708119629@163.com (J.W.); menke@scu.edu.cn (K.M.); 2 Department of Pharmacy, Personalized Drug Therapy Key Laboratory of Sichuan Province Sichuan Academy of Medical Sciences & Sichuan Provincial People’s Hospital, School of Medicine, University of Electronic Science and Technology of China, Chengdu 610072, China; shuaiming1998@163.com

**Keywords:** siRNA, p65, rheumatoid arthritis, gene therapy, micelle

## Abstract

Rheumatoid arthritis (RA) is one of the most common autoimmune diseases worldwide, causing severe cartilage damage and disability. Despite the recent progress made in RA treatment, limitations remain in achieving early and efficient therapeutic intervention. Advanced therapeutic strategies are in high demand, and siRNA-based therapeutic technology with a gene-silencing ability represents a new approach for RA treatment. In this study, we created a cationic delivery micelle consisting of low-molecular-weight (LMW) polyethylenimine (PEI)–cholesterol–polyethylene glycol (PEG) (LPCE) for small interfering RNA (siRNA)-based RA gene therapy. The carrier is based on LMW PEI and modified with cholesterol and PEG. With these two modifications, the LPCE micelle becomes multifunctional, and it efficiently delivered siRNA to macrophages with a high efficiency greater than 70%. The synthesized LPCE exhibits strong siRNA protection ability and high safety. By delivering nuclear factor kappa-light-chain-enhancer of activated B cells (NF-κB) p65 siRNA, the p65 siRNA/LPCE complex efficiently inhibited macrophage-based cytokine release in vitro. Local administration of the p65 siRNA/LPCE complex exhibited a fast and potent anti-inflammatory effect against RA in a mouse model. According to the results of this study, the functionalized LPCE micelle that we prepared has potential gene therapeutic implications for RA.

## 1. Introduction

Rheumatoid arthritis (RA) is one of the most common autoimmune diseases, with chronic arthritis as the main symptom, and it can cause cartilage and bone damage as well as disability [1]. With the growing understanding of the pathogenesis of this disease, some new therapeutics have emerged, leading to improved outcomes [2]. Current treatment strategies emphasize the need for early diagnosis and therapeutic intervention based on the use of disease-modifying anti-rheumatic or nonsteroidal anti-inflammatory drugs [3]. However, though progress has been made in the past several decades, a number of issues continue to persist. In particular, the development of advanced therapeutic strategies to achieve early and highly effective treatment to prevent the manifestation of RA is of great interest.

RNA silencing technology appeared as a potential game changer in therapeutics development [4,5,6,7]. Its rapid development has also led to the approval of three siRNA drugs by the United States Food and Drug Administration in recent years [8,9]. Regarding RA, research has already recognized that inflammation is closely related to its occurrence and progression. This inflammatory process is regulated by various cytokines released by immune cells and further leads to elevation of the inflammatory response by activating endothelial cells and recruiting more immune cells, leading to accumulation [10,11]. During RA, the infiltration of a large number of macrophages in synovial tissue initiates a variety of other immune cells, and this activated inflammatory cascade further results in cartilage and bone destruction [12,13,14]. Furthermore, as part of a family of inducible transcription factors, nuclear factor kappa-light-chain-enhancer of activated B cells (NF-κB) has been found to be a major inflammatory mediator of macrophages during RA; thus, it also mediates the induction of pro-inflammatory cytokines such as TNF-α, IL-1, and IL-6 [15,16]. Deregulated NF-κB activation contributes to the pathogenesis of inflammation [17]. As such, reversing its activity in macrophages during RA by siRNA-based gene silencing is believed to be a potential therapeutic strategy.

One of the main hurdles for developing siRNA-based gene therapeutics is the siRNA delivery vector. Transporting therapeutic siRNA into RA sites raises extra concerns. The main targets for siRNA-based RA therapy are immune cells, which are well known to be hard to gene-transfect [18,19]. Although macrophages are specialized in the phagocytosis of harmful antigens, gene delivery with non-viral vectors is still difficult [20]. The antigen processing and present nature of macrophages make them more sensitive to toxic substances such as exogenous organic compounds and positive charges. Further, the resulting cell death may attract more immune cells, thus ruining the whole treatment by elevating the immune response during RA. In addition, the large amounts of enzymes contained in lysosomes challenges the gene/vector complex with a high degradation risk, and this further prevents siRNAs from functioning [21]. For these reasons, siRNA-based RA gene therapy strategies are limited, despite the range of gene targets is widespread.

PEI25K (high-molecular-weight polyethylenimine (PEI)) is a classical cationic agent, and it can transfect different types of nucleic acids, so it is often called the “gold-standard” transfection agent. However, the abundant primary amino groups in PEI not only provide many positive charges for gene binding but also result in strong toxicity, limiting the further application of PEI as an appropriate gene-delivery vector [22]. In contrast, low-molecular-weight (LMW) PEI (PEI2K) possesses fewer positive charges and a poor gene-delivery ability. However, its high safety has attracted significant attention regarding developing it into a potential gene vector by cross-linking [23]. In the research of the past few years, PEI2K has been treated as a functional group in most studies, with few studies treating it as a compound core, and its potential as a cationic backbone has thus been ignored. LMW PEI has a slightly positive charge and is rich in amino acids, which can provide more possibilities and flexibility if introduced into functional modifications, hence it may also act as a potential carrier [24]. More importantly, novel gene vectors based on modified LMW PEI could achieve an appropriate balance of efficiency, safety, and stability, thus facilitating RA treatment [25].

Here, we developed a novel LMW PEI–cholesterol–PEG (LPCE) delivery micelle for RA gene therapy based on NF-κB p65 siRNA. This carrier is based on LMW PEI and modified with cholesterol and PEG. Due to these two modifications, the LPCE micelle becomes multifunctional. Furthermore, considering the important role of NF-κB in macrophages during RA, we chose NF-κB p65 siRNA to complex with the LPCE micelle. NF-κB p65 is a eukaryotic transcription factor, playing an important role in regulating multiple biological functions including inflammation, immunity, cell proliferation, and apoptosis [26]. We believe that our LPCE micelle could efficiently deliver p65 siRNA into macrophages in vivo, resulting in an early and fast anti-inflammation effect at the articular cavity with high safety. In this work, the siRNA delivery and protection abilities of the LPCE micelle were characterized in detail. In addition, we examined the treatment effects and mechanisms of p65 siRNA/LPCE in a collagen-induced arthritis (CIA) model in vitro and in vivo.

## 2. Methods

### 2.1. Preparation of LPCE Micelle

For the synthesis of the LPCE polymer, chloroform was used as a solvent to dissolve the branched PEI1.8K. After stirring at room temperature for 20 min, cholesterol chloroformate and PEG (molecular weight 550 Da) were slowly added to the solution. Once they were completely dissolved, the solution was transferred to an addition funnel. Then, this mixture was slowly dropped into the PEI solution, while continuing to stir. After rotary evaporation, ethyl acetate was added to dissolve the remaining material, then n-Hexane was added to precipitated and ultrapure water was used for rehydrating. Finally, the aqueous solution was freeze-dried, and the final product of LPCE polymer was obtained. The LPCE micelle solution was further prepared by dissolving LPCE in water at room temperature. The particle size and zeta potential of LPCE were determined using a Malvern sizer (Malvern, Mervyn, UK). Using transmission electron microscopy (TEM) (H-6009IV, Hitachi, Tokyo, Japan), we observed the morphology of the LPCE cationic micelles in detail. All results were the mean value of three tests.

### 2.2. siRNA Gel-Retarding Assay

LPCE micelles with different molecular proportions of NF-κB p65 siRNA were used to evaluate the binding ability of siRNA. NF-κB p65 siRNA (0.5 μg) mixed with LPCE at different ratios was electrophoresed on a 1% (*w*/*v*) agarose gel for 15 min at 120 V. When LPCE binds to siRNA, the molecular weight increases and migration in the gel is prevented, so that the bands appear to be shortened. GoldView II Nuclear Staining Dye (Solarbio, Beijing, China) was added to the gel, and the strips were examined using an E-gel imager (Bio-Rad, ChemiDox XRS, Hercules, CA, USA).

### 2.3. In Vitro Transfection

Murine macrophage RAW264.7 cells and 293T cells were incubated to test the transfection ability of LPCE. We incubated mouse macrophage RAW264.7 cells and 293T cells in 24-well plates for 24 h. Then, 1 μg of FAM siRNA and 5 μg of LPCE micelle were mixed in Dulbecco’s Modified Eagle Medium (DMEM) and added to each well. Four hours later, this medium was replaced with full medium; after another 24 h, the cell fluorescence was observed under a microscope, and the transfection efficiency was determined using a NovoCyte Flow Cytometer (ACEA Biosciences, San Diego, CA, USA). PEI25K/FAM siRNA complex (1:1, *w*/*w*) and bare FAM siRNA were used as controls.

### 2.4. Ribonuclease (RNase)-Degradation Assay

To study the ability of LPCE to protect siRNA against RNase A (Solarbio, Beijing, China), naked NF-κB p65 siRNA and NF-κB p65 siRNA/LPCE were exposed to RNase A at a final concentration of 0.25 mg/mL. Naked NF-κB p65 siRNA and NF-κB p65 siRNA/LPCE were exposed to the RNase A for 0 and 15 min and for one, two, and four hours, respectively. Then, sodium dodecyl sulfate (SDS) (0.24 mg/mL) was added for denaturation at 70 °C for 10 min. In addition, heparin (1 mg/mL) was added to NF-κB p65 siRNA/LPCE and the mixture placed on ice for five minutes to extract siRNA. Finally, all samples were examined by electrophoresis on a 1% (*w*/*v*) agarose gel for 15 min at 120 V.

### 2.5. Quantitative Real-Time Polymerase Chain Reaction (PCR)

NF-κB p65 siRNA (sense, 5′-GCG ACA AGG UGC AGA AAG ATT-3′; anti-sense, 5′-UCU UUC UGC ACC UUG UCG CTT-3′), FAM-labeled siRNA, and scramble siRNA were purchased from GenePharma Co., Ltd. (Suzhou, China). The silencing effect against the NF-κB p65 gene in RAW264.7 cells was studied using real-time quantitative PCR. Briefly, we first incubated mouse RAW264.7 cells in 24-well plates for 24 h, then 5 μg of LPCE micelle and 1 μg of FAM siRNA were dissolved in DMEM and added to each well. Four hours later, we removed the DMEM from the pores and replaced it with full medium. After another 24 h, TRIzol reagent (Vazyme Biotech Co., Ltd., Nanjing, China) was used to isolate total RNAs from transfected RAW264.7 cells. In addition, a SuperScript II Reverse Transcriptase kit (Takara Bio, San Jose, CA, USA) was used to synthesize complementary DNA, and an SYBR GreenER quantitative PCR SuperMix Universal kit (Thermo Fisher, Waltham, MA, USA) was used for real-time quantitative PCR. After running a standard cycling routine, reactions were carried out on an AB7500 real-time PCR system. The PCR primers to detect the level of NF-κB p65 (forward, GAC CTG CTG CTT CTG TGT CT; reverse, TCG TCA TCC TTA AGG GCC TG) and TNF-α (forward, CCC AGG GAC CTC TCT CTA ATC; reverse, ATG GGC TAC AGG CTT GTC ACT) were synthesized and purified by Tsingke Biological Technology (Chengdu, China).

### 2.6. Validation of M1/M2-Type Macrophages

M1 and M2 are two known polarization states of macrophages. To verify the effect of p65 siRNA/LPCE on the polarization state of macrophages, we firstly cultured a 24-well plate of RAW264.7 cells (1.5 × 10^5^ cell/well). Twelve hours later, LPS (lipopolysaccharide) at a concentration of 1 mg/mL was added to the wells for eight hours. Then, 1 μg of p65 siRNA/LPCE (p65 siRNA:LPCE = 1:5) was added to the hole with DMEM for four hours. After cells were incubated in incubator for 24 h, all cells were collected from cell pore plates for flow cytometry. Briefly, the cell suspension and adherent cells were collected for flow cytometry, and the CD206 antibody was incubated for 30 min. The expression level of CD206 in cells was detected by flow cytometry.

### 2.7. Cytotoxicity Assay

To detect the in vitro cytotoxicity of LPCE micelles, an MTT assay was used. Briefly, 293T cells were plated in 96-well plates (5 × 10^3^ cells/well) overnight at 37 °C. When the number of cells was as required, different concentrations of LPCE were added to the cell plates. PEI25K was used as a standard control. After transfecting for 24 h at 37 °C, 20 μL of MTT solution was added to stop the reaction, and the cells were incubated for another four hours. Subsequently, the medium in the plate was replaced with 150 μL of dimethyl sulfoxide, and the plates were shaken on a shaker for 5 to 15 min. Finally, the absorbance of each hole was measured at 570 nm using a SpectraMax absorbance reader (Molecular Devices, San Jose, CA, USA).

### 2.8. Cellular Uptake Mechanism of the siRNA/LPCE Complex

Different inhibitors of internalization pathways were used to determine the cellular uptake mechanism. Briefly, RAW264.7 cells were plated in 24-well plates (8 × 10^4^ cells per well) for 24 h, then pretreated with different inhibitors including amiloride (3 mM), filipin ш (4 μg/mL), chlorpromazine hydrochloride (1 μg/mL), and methyl-β-cyclodextrin (8.3 mM) for 30 min. These concentrations of inhibitors do not produce cytotoxicity. Following treatment with the various inhibitors, the cells were transfected with FAM siRNA/LPCE (1:5, *w*/*w*) for 24 h, and flow cytometry was used to determine the transfection efficiency rate. For fluorescent staining, 8 × 10^4^ cells were seeded in chamber slides (four-well glass; Millicell, St. Louis, MO, USA) and cultured overnight. Then, the RAW264.7 cells were treated with different inhibitors according to the previous results concerning the inhibitory effect. After transfecting for 24 h, nuclei were stained with Hoechst stain (1 mg/mL; Solarbio), and plasma membranes were stained with Dil stain (10 mg/mL, Beyotime Biotechnology, Haimen, China). The resultant fluorescence was observed and photographed using fluorescence microscopy (ZEN 880; Bamboo Living, Jena, Germany).

### 2.9. Detection of IL-10 Expression Level by Enzyme-Linked Immunosorbent Assay (ELISA)

To detect the concentration of the expressed IL-10 cytokine after stimulating with lipopolysaccharide (LPS), the cell culture supernatant of RAW264.7 cells was investigated using ELISA. Firstly, RAW264.7 cells were fostered into a six-well plate with a density of 3 × 10^5^ cells per well; then, 1 μg/mL of LPS was added to each well for 12 h. After that, NF-κB p65 siRNA/LPCE complex was added to each well (NF-κB p65 siRNA, 2 μg/well; siRNA/LPCE, 1:5, *w*/*w*) and incubated for four hours. At 72 h post-transfection, the cell culture supernatant was collected and concentrated. According to the operating instructions, a mouse IL-10 ELISA kit (Thermo Fisher Scientific, Waltham, MA, USA) was used to detect the cytokine level of anti-inflammatory factor IL-10.

### 2.10. Animal Study

All animal procedures were approved and controlled by the Institutional Animal Care and Treatment Committee of Sichuan University. To generate the CIA model, seven- or eight-week-old male DBA/1 mice were immunized intradermally with bovine CII (100 µg; Sigma-Aldrich, St. Louis, MO, USA) emulsified in CFA (Difco Laboratories, Franklin Lakes, NJ, USA). Mice were re-challenged with CII in IFA (Difco Laboratories) 21 days later and, on day 28, most showed features of CIA. According to the principle of random grouping, 15 mice were divided into the following five groups: (1) normal, (2) PBS, (3) LPCE, (4) scramble siRNA/LPCE, and (5) NF-κB p65 siRNA/LPCE. In the treatment group, intra-articular administration of NF-κB p65 siRNA/LPCE (siRNA = 10 μg/mouse, siRNA:LPCE = 1:6) was completed once every three days, for a total of 10 times, with the same dose of PBS, LPCE, and scramble siRNA/LPCE in the control groups. In addition, the mice in the normal group were not treated at all. The development of joint inflammation in the mice was examined daily, and scores were awarded as follows: zero points, normal; one point, redness and slight swelling confined to the tarsal joints or ankle; two points, erythema and mild swelling from the ankle to the metatarsal or metacarpal joints; three points, erythema and moderate swelling beginning at the ankle to the metatarsophalangeal or metacarpophalangeal joints; four points, erythema and severe swelling can be found in the entire area from the ankle to the toe of the mouse. The highest score for arthritis for each mouse was 4 points, and the highest score for disease was 16 points. The PRISM version 3.0 algorithm (GraphPad Software, La Jolla, CA, USA) was used to calculate the average clinical score for all animals in the group. The body weights of mice were also measured on the same days. On day 30 of administration, all mice were sacrificed, and limbs were isolated for further analysis.

### 2.11. Histological Examination

On day 30 after treatment, the mice were killed, and their joint tissues were harvested. The tissues were fixed with 10% formalin/phosphate-buffered saline (PBS), then decalcified with ethylenediaminetetraacetic acid for 21 days and embedded in paraffin. The paraffin-embedded tissue sections were dewaxed and rehydrated, then stained with immunehistochemical and hematoxylin and eosin (H&E). The hydrated sections were stained with H&E. After the H&E staining of joint sections, ImageJ software (National Institutes of Health, Bethesda, MD, USA) was used to analyze the tissue from inside the joints. To detect NF-κB p65 and F4/80 in the tissue, after blocking, joint sections were incubated with corresponding rabbit anti-mouse primary antibodies (Abcam, Cambridge, UK) in a refrigerator at 4° overnight. The appropriate horseradish peroxidase-conjugated secondary antibody was then applied. Using fluorescence microscopy (Olympus Corp., Tokyo, Japan), these markers were observed and determined.

### 2.12. Cytokine Levels Analysis In Vivo

To analyze the cytokine levels in the joints of mice, RNA was extracted from the synovial membranes of the ankle joints of the mice after they were killed. Firstly, we cut off the ankle joint of the mouse, removed the synovial membrane of the joint, and placed it in a cryo-storage tube precooled with liquid nitrogen. The synovium of the joint was removed and ground into powder using a mortar and pestle. Then, total RNA was extracted using TRIzol reagent (Thermo Fisher Scientific, Waltham, MA, USA), and the expression level of cytokines was detected using real-time PCR. PCR primers were used to detect the levels of arthritic factor NF-κB p65 (forward, GAC CTG CTG CTT CTG TGT CT; reverse, TCG TCA TCC TTA AGG GCC TG), IFN-γ (forward, CTA ATT ATT CGG TAA CTG ACT TGA; reverse, ACA GTT CAG CCA TCA CTT GGA), IL-1β (forward, ACA GAT GAA GTG CTC CTT CCA; reverse, GTC GGA GAT TCG TAG CTG GAT), IL-6 (forward, GCA AAG GGA AAC TCA CCA; reverse, AAC ACC TGT TTG GCT TTT AG), TNF-α (forward, CCC AGG GAC CTC TCT CTA ATC; reverse, ATG GGC TAC AGG CTT GTC ACT), and IL-10 (forward, CAT CGA TTT CTT CCC TGT GAA; reverse, TCT TGG AGC TTA TTA AAG GCA TTC).

### 2.13. Statistical Analysis

Data are presented as averages with 95% confidence intervals. All data were statistically analyzed using PRISM version 5.0c software (GraphPad Software, La Jolla, CA, USA). Statistical significance was assigned at values of *p <* 0.05 (95% confidence interval).

## 3. Results

### 3.1. Preparation and Characterization of LPCE Micelles

**Scheme 1:**
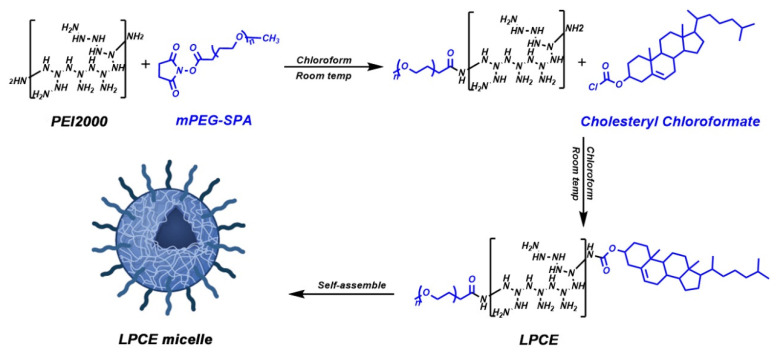
Preparation process of LPCE micelle.

**Figure 1:**
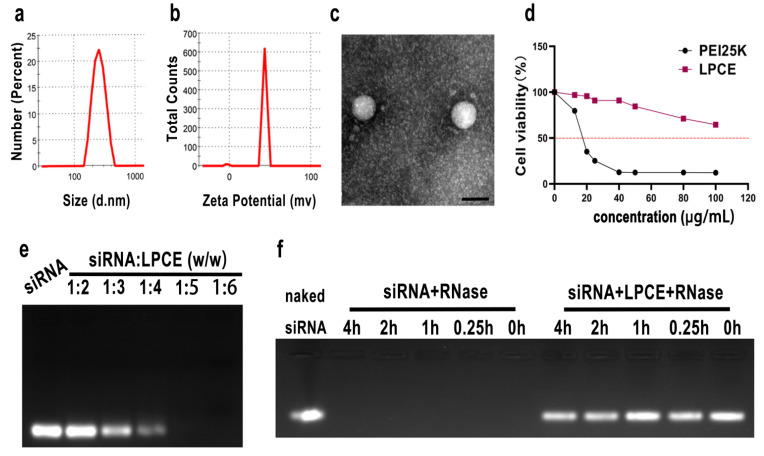
Characterization of LPCE micelle. (**a**,**b**) Size distribution and zeta potential of the LPCE. (**c**) Transmission electron microscopy photomicrographs of LPCE (scale bar, 200 nm). (**d**) Cell viability assay of LPCE and PEI25K on 293T cells. (**e**) Gel-retarding assay of the LPCE/p65 siRNA complex. (**f**) Ribonuclease protection assay of the LPCE/p65 siRNA complex.

The cationic LPCE micelles were prepared based on LMW PEI and modified with cholesterol and PEG (Scheme 1). The synthesized LPCE formed a clear solution when dissolved in water. The dynamic diameter of the cationic micelles was 334.2 ± 4.4 nm, with a polydispersity index of 0.42. The zeta potential was 43.4 ± 0.8 mV (Figure 1a,b). The morphology of LPCE micelles was observed using transmission electron microscopy. Transmission electron microscopy measurements (Figure 1c) showed that the prepared LPCE micelles were mono-dispersed and spherical, with a particle size close to 100 nm, which was consistent with the abovementioned particle size evaluation results. These results indicate that LPCE can self-assemble into cationic micelles in aqueous solution, which is an important capability for use in delivering siRNA. The loading potential of LPCE for siRNA was verified by an siRNA delay experiment. As shown in Figure 1e, when the ratio of siRNA to LPCE reached 1:5 (*w*/*w*), the siRNA bands disappeared completely, i.e., the siRNA was completely bound with positively charged LPCE micelles through electron complexation. Therefore, other relevant experiments were subsequently carried out using this ratio. The capacity to protect siRNA from enzymatic degradation is an important property of a transfer vector, and we performed ribonuclease degradation tests to verify this ability. As shown in Figure 1f, after complexing with LPCE, the siRNAs could effectively prevent enzymatic degradation for up to four hours. Bands of siRNA extracted from the siRNA/LPCE complex were as bright as bare siRNA and showed strong protection. The cytotoxicity of the LPCE micelles for 293T cells was detected by MTT assay. According to our results, the IC_50_ of LPCE micelles was greater than 100 μg/mL, while that of PEI25K was only 15 μg/mL (Figure 1d), indicating the high safety of the prepared LPCE micelles.

### 3.2. LPCE Could Deliver siRNA Safely and Efficiently

**Figure 2:**
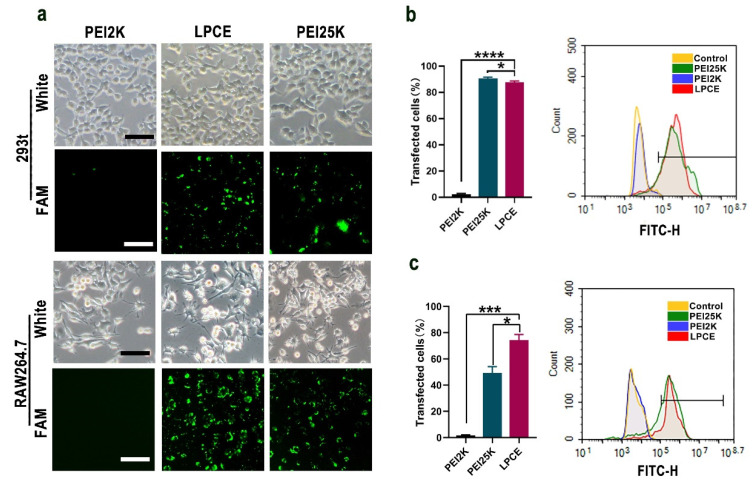
In vitro gene transfection study of LPCE micelle. (**a**) Fluorescence microscopy images of RAW264.7 and 293T cells (scale bar, 100 μm). FAM is fluorescently labeled siRNA (**b**) Transfection efficiency of LPCE micelle on 293T cells (**** *p* < 0.0001, * *p* < 0.05) analyzed by flow cytometry. (**c**) Transfection efficiency of LPCE micelle on RAW264.7 cells (*** *p* < 0.001, * *p* < 0.05) analyzed by flow cytometry.

RAW264.7 cells and 293T cells were used to test the siRNA delivery capacity of the prepared LPCE micelles. As shown in Figure 2a, the LPCE micelles could effectively transfer FAM reporting siRNA into RAW264.7 cells and 293T cells with good safety (FAM siRNA, 1 μg/well; siRNA LPCE, 1:5, *w*/*w*). As calculated by flow cytometry, the transfection efficiency of LPCE in 293T cells reached 89.2% (*p* < 0.1 vs. the PEI25K group; *p* < 0.001 vs. the PEI2K group), while this value for PEI25K was 91.41% (Figure 2b). In RAW264.7 cells, the transfection rate of the LPCE complex reached 74.36% (*p* < 0.01 vs. the PEI25K group; *p* < 0.001 vs. the PEI2K group), which was much higher than that of the gold-standard transfection agent PEI25K (49.26%) (Figure 2c). Furthermore, the transfection capacity of PEI2K (2.1%) was very low, much lower than that of LPCE, indicating that the functional modification of LPCE did promote its siRNA delivery capacity. Therefore, our results suggested that LPCE showed high efficiency for siRNA-based transfection into RAW264.7 cells and 293T cells.

### 3.3. Cellular Uptake Mechanism Study of the siRNA/LPCE Complex

**Figure 3:**
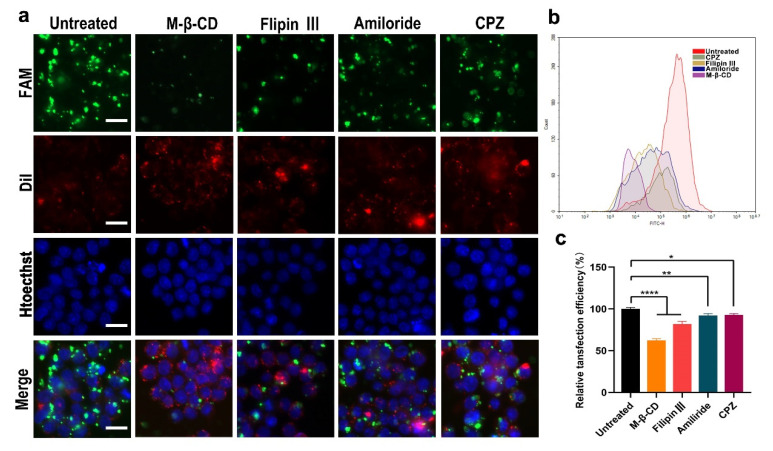
Cell uptake mechanism of the siRNA/LPCE complex. (**a**) Fluorescent image of FAM siRNA/LPCE complex pretreated with different inhibitors. The transfection efficiency in the methyl-β-cyclodextrin treatment group was obviously reduced, FAM is fluorescently labeled siRNA and Dil was used to stain the nucleus and Hoechst was used to stain the cell membrane (scale bar, 25 μm). (**b**,**c**) The uptake rates calculated by flow cytometry (* *p* < 0.05, ** *p* < 0.01, **** *p* < 0.0001).

As can be seen from the above experimental results, the LPCE system has a strong siRNA delivery capacity; hence, we studied the cell uptake mechanism of siRNA delivered by LPCE. Four endocytosis pathways including reticular-protein-mediated endocytosis, lipid-raft-mediated endocytosis, caveolin-mediated endocytosis, and endocytosis are believed to play important roles in the uptake of cargoes. We investigated whether certain internalization pathways were activated during the uptake process of the siRNA/LPCE complex. To verify this, we pretreated RAW264.7 cells with different inhibitors of the above pathway before transfection: lipid-raft-mediated endocytosis (methyl-β-cyclodextrin), cage-protein-mediated endocytosis (chlorpromazine hydrochloride), pit-protein-mediated endocytosis (filipin III), and pinocytosis (amiloride). As shown in Figure 3a, the transfection efficiency in the methyl-β-cyclodextrin treatment group was obviously reduced compared to that in the other treatment groups. As detected by flow cytometry, an average inhibition rate of 64.6% (*p* < 0.0001 vs. the control group) was calculated in the methyl-β-cyclodextrin treatment group (Figure 3b,c). These results suggested that, through lipid-raft-mediated endocytosis, NF-κB p65 siRNA/LPCE was taken in specifically. These results effectively explain why LPCE had such a good siRNA delivery capacity.

### 3.4. NF-κB p65 siRNA/LPCE Complex Efficiently Regulated Macrophage Activities In Vitro

**Figure 4:**
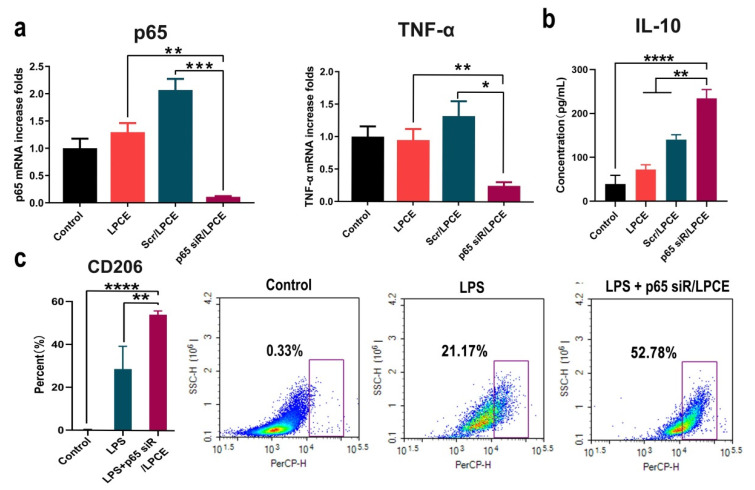
In vitro anti-inflammatory study of p65 siRNA/LPCE complex. (**a**) The levels of p65 mRNA and tumor necrosis factor alpha messenger RNA in RAW264.7 cells after transfection (*** *p* < 0.001, ** *p* < 0.01, * *p* < 0.05). (**b**) The interleukin-10 level detected by enzyme-linked immunosorbent assay in the supernatant of macrophages (**** *p* < 0.0001, ** *p* < 0.01). (**c**) The expression of CD206 by flow cytometry in the p65 siRNA/LPCE-treated and untreated groups after stimulation by LPS.

To investigate the inhibitory effect of LPCE-mediated NF-κB p65 siRNA on arthritis, we conducted an in vitro study. First, RAW264.7 cells were transfected with NF-κB p65 siRNA/LPCE (siRNA, 1 μg/well; LPCE, 5 μg/well); then, its ability to silence NF-κB p65 expression was evaluated using real-time PCR. As shown in Figure 4a, after 24 h, the NF-κB p65 mRNA level in the NF-κB p65 siRNA/LPCE group was obviously decreased (*p* < 0.001 vs. the scramble siRNA/LPCE group; *p* < 0.01 vs. the LPCE group) compared with that in the control group, and the TNF-α mRNA level in the NF-κB p65 siRNA/LPCE group was also decreased (*p* < 0.05 vs. the scramble siRNA/LPCE group; *p* < 0.01 vs. the LPCE group) compared with that in the control group (Figure 4a). It is well known that the secretion of TNF-α is regulated by NF-κB p65, and TNF-α, which is more prominent than other inflammatory factors, is also known to influence arthritis. Therefore, the above experimental results showed a high level of siRNA delivery efficiency and effective silencing of the genes involved by NF-κB p65 siRNA. Furthermore, as shown in Figure 4b, after 12 h of LPS stimulation and transfection of NF-κB p65 siRNA/LPCE, a high level of anti-inflammatory factor IL-10 could be detected via ELISA (*p* < 0.0001 vs. the control group; *p* < 0.01 vs. the scramble siRNA group). LPS is an endotoxin that can induce a strong immune response. Under the stimulation of LPS, macrophages differentiate into M1-type macrophages. IL-10 is an important anti-inflammatory factor regulated by the NF-κB pathway and is mainly secreted by M2 macrophages. The observed increased expression of IL-10 demonstrated that NF-κB p65 siRNA could effectively inhibit the endogenous and upregulated expression of NF-KB p65 in RAW264.7 cells after LPS stimulation. To further validate this, we then activated RAW264.7 cells into the M1 type using LPS before treatment with the p65 siRNA/LPCE complex. According to our results (Figure 4c), the number of M2-type cells was significantly increased after treating the cells for 24 h with p65 siRNA/LPCE. After the same LPS stimulation, CD206 expression increased in the siRNA-treated group compared with the untreated group (*p* < 0.01). CD206 is the specific CD antigen of M2 macrophages; a high expression of CD206 means that more cells in the p65 siRNA/LPCE-treated group were polarized to M2. It was found that p65 siRNA/LPCE had the ability to polarize M1-type macrophages to M2-type macrophages. Therefore, we proved that NF-κB p65 siRNA was successfully delivered and expressed in RAW264.7 cells with the help of the LPCE system, providing efficient support for further anti-arthritic activity.

### 3.5. NF-κB p65 siRNA/LPCE Complex Efficiently Suppressed Arthritis Development In Vivo

**Figure 5:**
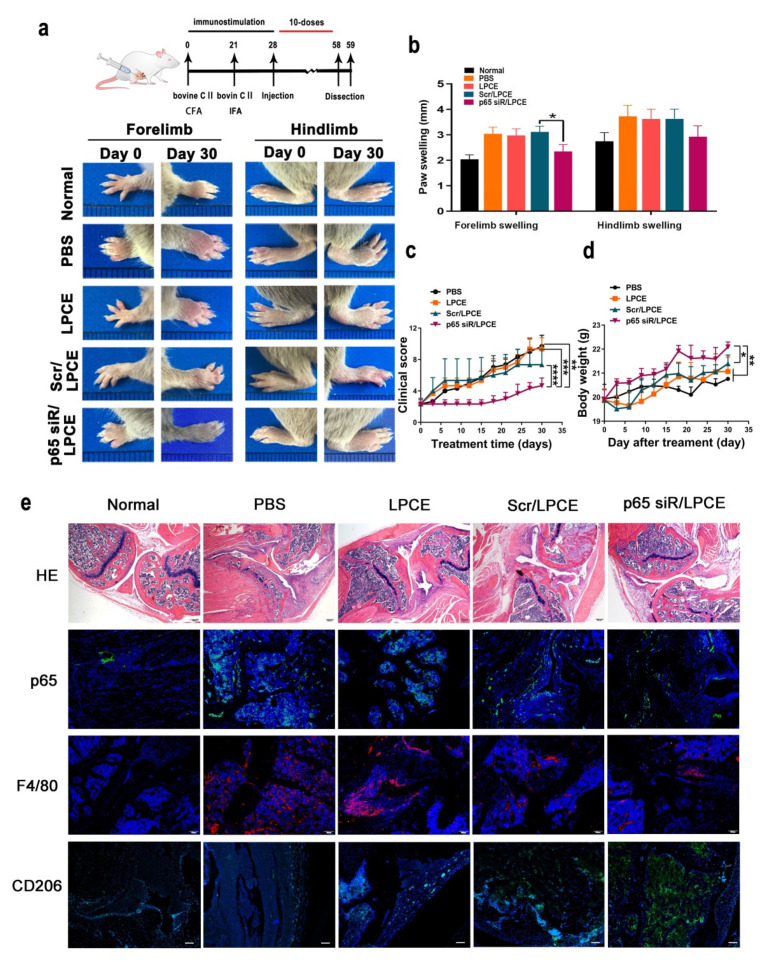
The p65siRNA/LPCE complex inhibits disease development and ameliorates pathological damage in collagen-induced arthritis mice. (**a**) Arthritic images of the hind and forelimbs. (**b**) Paw-swelling measurements taken during the period of treatment. (**c**) Clinical score measurements taken during treatment. (**d**) Body weight measurements taken during treatment. Values are presented as mean ± standard error of the mean (*n* = 5 per group) (**** *p* < 0.0001, *** *p* < 0.001, ** *p* < 0.01, * *p* < 0.05). (**e**) H&E and immunohistofluorescence staining. Scale bar, 100 μm.

In order to simulate the arthritis process, we established a mouse CIA model. This model is established through the use of bovine CII intradermal immunity. It is a particularly important model that can be used to study inflammation. Throughout the growth and development of the mice, monitoring took place to verify the in vivo effects of the NF-κB p65 siRNA/LPCE complex. As shown in Figure 5a, after ensuring that all model mice in the experimental group became ill, we monitored the progression of the CIA for one month from the day of the first treatment. On day 0 of the treatment, the tarsal joints of the mice in the treatment group were slightly swollen, and the swelling and erythema gradually increased with time. As the treatment continued, the arthritis scores of the paws of the mice in the PBS-treated and LPCE-treated groups gradually increased, while in the scramble siRNA/LPCE group, the disease development of the mice was relatively slow. In addition, the arthritis score of mice treated with the NF-κB p65 siRNA/LPCE micelle was almost completely blocked. It is worth noting that we found that the NF-κB p65 siRNA/LPCE-treated group experienced an effect beginning on the third day; from the third day of administration, the course of the disease progressed more slowly in the NF-κB p65 siRNA/LPCE group. On day 30, the average clinical scores of the PBS-, LPCE-, and scramble siRNA/LPCE–treated groups had increased, and all these groups developed severe arthritic inflammation. The mean clinical scores of the PBS- and LPCE-treated groups increased fourfold, and the average clinical score of the scramble siRNA/LPCE–treated group increased threefold, whereas there was no significant trend in the clinical mean scores of the NF-κB p65 siRNA/LPCE group (Figure 5d). In addition, during the 30 days of treatment, the NF-κB p65siRNA/LPCE treatment group showed normal body weight development to a degree that was infinitely greater than that in the PBS-, LPCE-, or scramble siRNA/LPCE-treated groups (Figure 5c). Furthermore, we measured the thickness of the forelimbs and hindlimbs of the mice every day. As shown in Figure 5b, the paw swelling of mice in the NF-κB p65 siRNA/LPCE-treated group was the least severe. This was further evidence of the NF-κB p65 siRNA/LPCE complex’s powerful therapeutic effects.

As the H&E staining assay showed (Figure 5e), the synovium between the bones was intact in the NF-κB p65 siRNA/LPCE treatment group and there was no obvious synovial hyperplasia, cartilage damage, lymphocyte infiltration, or bone erosion, i.e., it was almost normal. In contrast, the PBS and LPCE treatment groups showed severe cartilage and bone erosion, with almost no complete joint cavity; in fact, histopathology and necrosis were extensive. In the scramble siRNA/LPCE treatment group, the degree of cartilage damage was slightly less, and the infiltration of inflammatory cells and the formation of keloids were slightly improved compared with the other groups. These findings indicated that NF-κB p65 siRNA/LPCE effectively inhibited the development of CIA and ameliorated disease severity.

Via immunofluorescence staining (Figure 5e) of the histology cross sections of arthritic joints, we could determine that downregulation of NF-κB p65 occurred in the in vivo system. In the NF-κB p65 siRNA/LPCE-treated group, the fluorescent signals of NF-κB p65 were significantly reduced in highly cell-dense regions of synovium in the CIA mice. F4/80 may also be used as a macrophage marker for immunohistochemical analysis of arthritic joints. As we can see, the expression of macrophages in mouse joint tissue increased in all treatment groups, but in the NF-κB p65 siRNA/LPCE-treated group, the fluorescent signals of NF-κB p65 were the lowest. In other words, macrophage expression was lowest in the mice of the NF-κB p65 siRNA/LPCE treatment group, thus corroborating the above findings that NF-κB p65 siRNA/LPCE complexes targeted macrophages and suppressed the NF-κB-dependent inflammatory response in the mitigation of clinical arthritis. Furthermore, in the immunofluorescence staining of CD206, we found that the expression of CD206 in the NF-κB p65 siRNA/LPCE group was significantly increased compared with the other groups. It was verified that after NF-κB p65 siRNA/LPCE treatment, the M2 type of mouse macrophages increased. An increase in M2 type macrophages promotes tissue repair and homeostasis recovery. The above immunofluorescence results confirmed that the NF-κB p65 siRNA/LPCE complex can effectively alleviate arthritis.

### 3.6. NF-κB p65 siRNA/LPCE Regulates Inflammatory Cytokine Production

**Figure 6:**
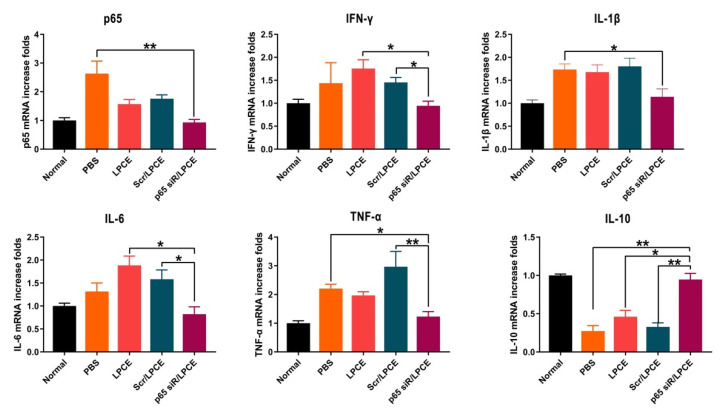
The p65 siRNA/LPCE complex regulates the secretion of inflammatory factors in vivo. The expression of anti-inflammatory cytokine IL-10 was significantly increased, and pro-inflammatory cytokine NF-κB p65, TNF-α, IFN-γ, IL-1β, and IL-6 were decreased. (** *p* < 0.01, * *p* < 0.05).

The secretion of inflammatory cytokines regulates the production and migration of inflammatory cells. Pro-inflammatory cytokines play an important role in CIA pathogenesis. We investigated the expression of NF-κB p65, TNF-α, IFN-γ, IL-1β, IL-6, and IL-10 in the synovium of mouse joints and found, in parallel with improved clinical indices and histology, that joint-associated inflammatory cytokine production was significantly regulated in mice treated with NF-κB p65 siRNA/LPCE micelles. As is shown in Figure 6, the concentrations of NF-κB p65, TNF-α, IFN-γ, IL-1β, and IL-6 mRNA were significantly elevated in the PBS, LPCE, and scramble siRNA/LPCE treatment groups, and there was no significant increase in the NF-κB p65 siRNA/LPCE treatment group. In contrast, the expression of anti-inflammatory cytokine IL-10 was significantly increased in the NF-κB p65 siRNA/LPCE treatment group. These early inflammatory factors are involved in the occurrence and development of the inflammatory response. The experimental results confirmed that the NF-κB p65 siRNA/LPCE micelle indeed regulates the expression of relevant inflammatory factors in the joint cavity, and that NF-κB p65 plays an important role in regulating the pathophysiological process of pro-inflammatory/anti-inflammatory imbalance. Silencing the expression of NF-κB p65 can effectively downregulate the inflammatory response.

## 4. Discussion

**Scheme 2:**
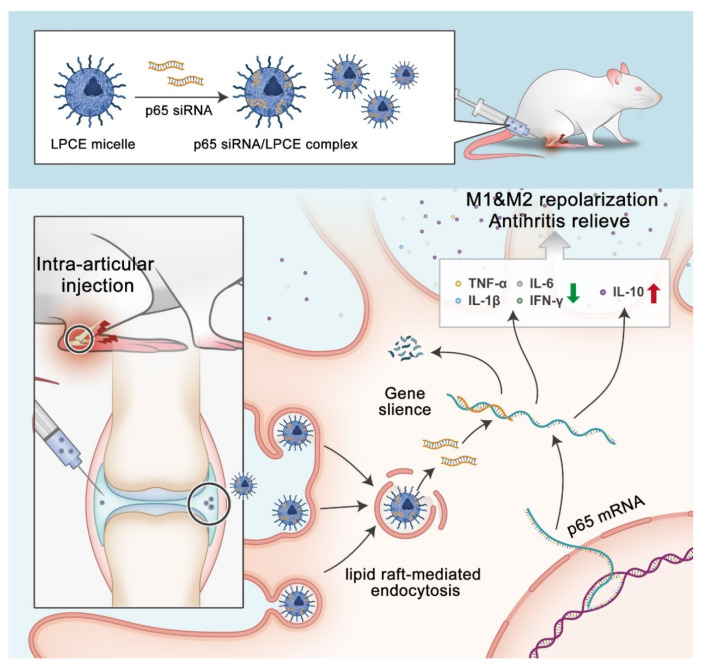
Therapeutic strategy and mechanism of p65siRNA/LPCE complex.

In this research, a new non-viral siRNA LPCE micelle vector was developed for siRNA-based RA gene therapy (Scheme 2). Our results demonstrated that the synthesized LPCE exhibits strong siRNA delivery efficiency and high safety. By delivering NF-κB p65 siRNA, the p65 siRNA/LPCE complex showed a therapeutic effect quite quickly and demonstrated strong arthritic inhibition ability in a CIA mouse model. Our results suggest that the LPCE functionalized micelle is an advanced candidate for siRNA-based RA gene therapy.

In almost all human cells, the RNA interference (RNAi) pathway regulates the stability and translation of mRNA. Owing to its capacity for silencing the mRNA levels of certain proteins in cells, siRNA-based gene therapy has been widely studied as a strategy for the treatment of several diseases. RNAi uses homologous double-stranded RNAs to induce silencing of sequence-specific target genes, rapidly blocking gene activity [27]. This method of gene silencing has become a new trend in anti-cancer therapy and anti-inflammatory therapy. The main hurdles in the development of therapeutics based on siRNA are poor stability, the low efficiency of siRNA delivery to target cells, and the degradation of siRNA by nucleases in biological fluids [28]. For example, Alexander et al. optimized PEI-based nanoparticles for siRNA delivery, then analyzed the results in in vitro and in vivo tumor tissue slice culture models [29]. Parmar et al. formulated an siRNA delivery system using lipid-substituted PEI and hyaluronic acid to inhibit growth and migration of triple-negative breast cancer cells [30]. To deliver siRNA into macrophages and B cells for BTK gene silencing, Zhao et al. employed a cationic lipid-assisted PEG-b-PLGA nanoparticle (CLAN) to encapsulate siRNA [31]. Finally, Zhen et al. designed a superparamagnetic iron oxide nanoparticle modified with PEI and galactose for siRNA targeted delivery in hepatocellular carcinoma therapy [32].

In recent years, the problem of how to coordinate the transfection efficiency and toxicity of PEI has been a huge challenge for researchers. In this research, we used LMW PEI as a potential vector, with functional modifications. It was found that this could provide more possibilities and greater flexibility [33]. In this way, additional functions could be obtained with controllable amino groups. This provides opportunities to reach an appropriate balance of efficiency and safety, as well as stability, thus facilitating RA treatment. Our results indicate that LPCE with a suitable particle size can potentially fulfill the demands for a competent siRNA delivery system, and siRNA can be efficiently delivered into cells with a high transfection efficiency. More importantly, LPCE is a single-vector system with a simple composition. We also showed that siRNA bound to LPCE was absorbed into cells through lipid-raft-mediated endocytosis, and LPCE protected siRNA from ribonuclease degradation. Furthermore, LPCE is a practical siRNA gene carrier with low toxicity. By loading the NF-KB p65 targeting siRNAs, the p65 siRNA/LPCE complex efficiently reduced the inflammatory progression of macrophages as planned.

RA is one of the most common autoimmune diseases with serious cartilage and bone damage symptoms. The exact cause of rheumatoid arthritis has not yet been fully established; according to existing literature reports, its pathogenesis involves multiple signaling pathways, and macrophages can severely drive RA progression by producing various pro-inflammatory factors, leading to joint destruction [34]. Key barriers to the successful development of RA treatment include a limited understanding of early cartilage degradation pathways and the ineffectiveness of therapeutic drug delivery to inherent chondrocytes without vascular cartilage. In previous studies, Frideriki et al. injected infliximab into the intraperitoneal cavity to treat CIA rat models [35], and Spieler et al. directed interleukin-4 to arthritic joints in a mouse model of antigen-induced arthritis [36]. In these studies, although certain therapeutic effects were achieved in the development of arthritis, there was still a lack of specific targets and treatment efficiency.

In our research, NF-κB was selected as a target for siRNA design. As is known, p65 is a member of the NF-κB family, and is a eukaryotic transcription factor playing an important role in regulating multiple biological functions including inflammation, immunity, cell proliferation, apoptosis, and tumor migration. In recent years, studies on NF-κB p65 have been increasing. For example, NF-κB p65 was found to be constitutively active in acute monocytic leukemia; therefore, studies have been conducted to regulate the proliferation and apoptosis of THP-1 cells in vitro and tumor growth in vivo by inhibiting the expression of NF-κB p65 for the treatment of acute monocytic leukemia [37]. Another study delivered p65 siRNA into the body through a glycerol-based branched-chain copolymer to downregulate the expression of p65 in A549 cancer cells and enhance apoptosis, thus enhancing cancer treatment [38]. As mentioned above, NF-κB p65 siRNA has been widely applied in various diseases, showing its powerful therapeutic effect, but there are few reports available to date on the application of NF-κB p65 siRNA in the treatment of arthritis.

In many inflammatory diseases, the NF-κB pathway plays a key role in the inflammatory response of macrophages, and the members of the NF-κB transcription factor family coordinate a wide range of stress-induced inflammatory responses and regulate developmental programs and cell differentiation. Members of the NF-κB transcription factor family orchestrate a wide range of stress-like inflammatory responses, regulate developmental programs and cellular differentiation, and control the growth and survival of normal and malignant cells [39]. In our research, we successfully transfected NF-κB p65 siRNA with LPCE micelles. The interference efficiency of siRNA was detected using real-time PCR and ELISA. The results showed that the introduction of siRNA significantly inhibited the NF-κB p65 of RAW264.7 cells at the gene level, indicating that the designed siRNA could effectively silence the expression of the target gene. In addition, NF-κB p65 siRNA can effectively inhibit endogenous and LPS-induced upregulated expression of NF-κB p65 in RAW264.7 cells, thus regulating the expression of anti-inflammatory factor IL-10. In the mouse CIA model, after p65 siRNA/LPCE treatment, the pro-inflammatory factors TNF-α, IL-1β, IFN-γ, and IL-6 in the synovial membrane of the articular cavity of the mouse were significantly decreased, and the anti-inflammatory factor IL-10 was significantly increased. It was confirmed that NF-κB p65 plays an important role in regulating the pathophysiological process of pro-inflammatory/anti-inflammatory imbalance. According to our results, when combined with a safe and effective micellar platform, NF-κB p65 siRNA is efficiently targeted and delivered to the arthritis site, avoiding off-target toxicity to a great extent, thereby specifically inhibiting arthritis. The p65 siRNA/LPCE complex effectively inhibited the expression of NF-κB p65 in macrophages, suppressing the release of pro-inflammatory factors. When administrated locally, the p65 siRNA/LPCE complex showed its therapeutic effect quite quickly and demonstrated strong arthritic inhibition ability in a CIA mouse model. Our results showed that the NF-κB p65 siRNA/LPCE micelle successfully regulates the expression of relevant inflammatory factors in the joint cavity. We also demonstrated that NF-κB p65 plays an important role in the pathophysiological process of pro-inflammatory/anti-inflammatory imbalance during RA. Silencing the expression of NF-κB p65 in macrophages via siRNA-based treatment is a potential strategy for RA gene therapy.
